# Gallium-68 fibroblast activation protein inhibitor positron emission tomography in cardiovascular disease

**DOI:** 10.3389/fnume.2023.1224905

**Published:** 2023-07-27

**Authors:** Dineo Mpanya, Mike Sathekge, Eric Klug, Jenna Damelin, Stuart More, Bawinile Hadebe, Mariza Vorster, Nqoba Tsabedze

**Affiliations:** ^1^ Division of Cardiology, Department of Internal Medicine, Faculty of Health Sciences, University of the Witwatersrand, Johannesburg, South Africa; ^2^ Department of Nuclear Medicine, University of Pretoria, Pretoria, Gauteng, South Africa; ^3^ Nuclear Medicine Research Infrastructure, Steve Biko Academic Hospital, Pretoria, South Africa; ^4^ Netcare Sunninghill, Sunward Park Hospitals, Johannesburg, South Africa; ^5^ Division of Nuclear Medicine, Department of Radiation Medicine, Faculty of Health Sciences, University of Cape Town, Cape Town, South Africa; ^6^ Department of Nuclear Medicine, College of Health Sciences, University of KwaZulu Natal, Durban, South Africa; ^7^ Department of Nuclear Medicine, Inkosi Albert Luthuli Central Hospital, Durban, South Africa

**Keywords:** gallium-68, fibroblast activation protein, positron emission tomography, cardiovascular disease, myocardial injury, fibrosis

## Abstract

Gallium-68 fibroblast activation protein inhibitor [(^68^Ga)Ga-FAPI] is a new radiopharmaceutical positioning itself as the preferred agent in patients with malignant tumours, competing with 2-Deoxy-2-[18F]fluoro-d-glucose [2-(^18^F)FDG] using positron emission tomography (PET). While imaging oncology patients with [^68^Ga]Ga-FAPI PET, incidental uptake of [^68^Ga]Ga-FAPI has been detected in the myocardium. This review summarises original research studies associating the visualisation of FAPI-based tracers in the myocardium with underlying active cardiovascular disease.

## 1. Introduction

Gallium-68 fibroblast activation protein inhibitor [^68^Ga]Ga-FAPI is a new radiopharmaceutical widely used when imaging patients with various malignancies, inflammatory and pre-fibrotic conditions. The tumour environment predominantly consists of cancer-associated fibroblasts (CAF) and non-malignant cells that play a role in cancer metabolism and regulate tumour growth and aggressiveness (1). These CAF overexpress the fibroblast activation protein, a binding site for [^68^Ga]Ga-FAPI. Similarly, increased fibroblast activation protein (FAP) expression in patients with cardiac disease has been identified at day seven post myocardial infarction (2). When a myocardial injury occurs, fibroblasts differentiate into myofibroblasts which produce components of the extracellular matrix (ECM), predominantly collagen, to allow healing and maintaining the structural integrity of the heart.

## 2. Gallium-68 fibroblast activation protein inhibitor positron emission tomography

**Figure 1:**
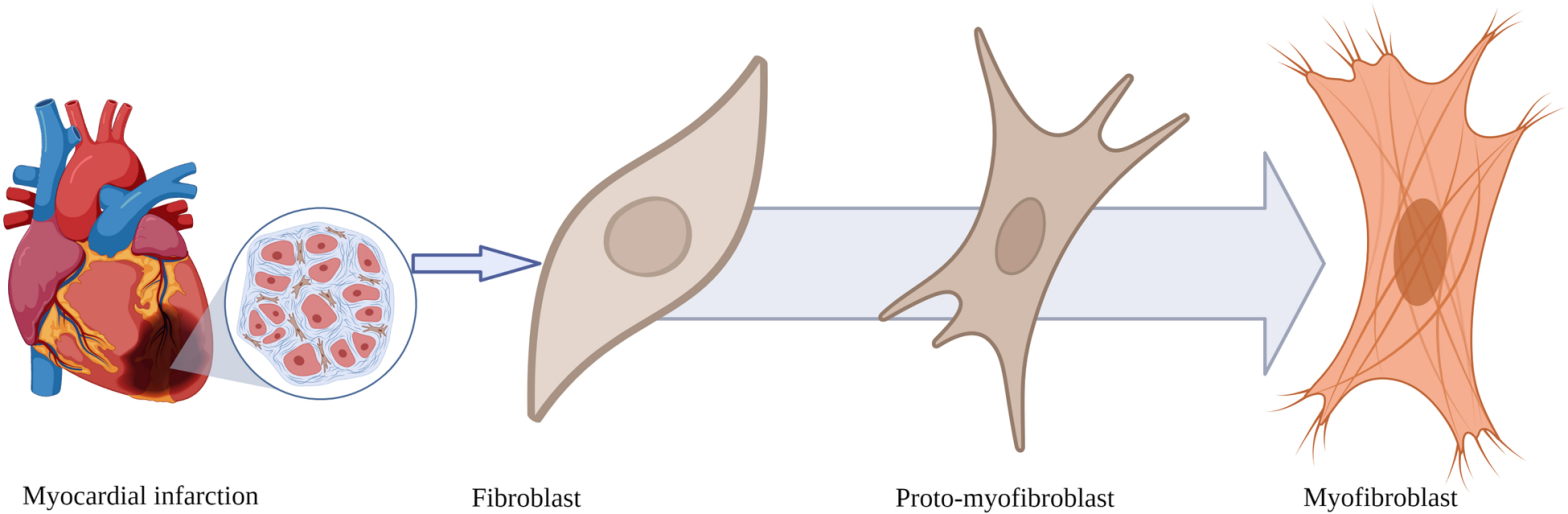
Morphological transformation of fibroblasts after myocardial infarction. Fibroblasts transform from a resting state into proto-myofibroblasts and myofibroblasts expressing fibroblast activation protein, the binding site for fibroblast activation protein inhibitor-based imaging agents.

Fibroblasts are elongated spindle-shaped cells with a basophilic cytoplasm, an oval nucleus, a well-developed Golgi apparatus, and an abundantly rough endoplasmic reticulum (Figure 1). Fibroblasts originate from mesenchymal cells derived from stem cells. They are the most abundant cell type in the connective tissue of various organs. The cellular membrane of activated fibroblasts expresses FAP, a transmembrane serine protease composed of amino acids with an intracellular domain of six amino acids, and a transmembrane domain of 20 amino acids (3).

Gallium-68 fibroblast activation protein inhibitor positron emission tomography (PET) has positioned itself as the preferred imaging modality for the staging and restaging of various oncological malignancies such as head, neck, and abdominal tumours, almost overthrowing 2-Deoxy-2-[^18^F]fluoro-d-glucose [2-(18F)FDG] PET (4). The ease of onsite preparation and the high tissue-to-background contrast ratio of gallium-labelled ligands render them ideal imaging agents over [2-(18F)FDG]. In some studies, [^68^Ga]Ga-FAPI PET has been used for radiation treatment planning and evaluation of biodistribution kinetics (5).

The synthesis of FAPI precursors for tumour binding and potential therapy, FAPI-02 up to FAPI-15, has been reported by Linder et al. (6). In their study, FAPI-04 had a favourable tumour-to-blood volume in patients with metastatic breast cancer. Also, FAPI-04 was more stable in human serum (6). Other FAPI precursors include FAP-34, which has been labelled with technetium-99m, FAPI-74, and FAPI-46 (7). Novel techniques for synthesising [^68^Ga]Ga-FAPI-46 involve mixing a buffer solution with ascorbic acid and 50 micrograms (µ) of FAPI-46 and transferring the mixture into the reactor vial. Gallium-68 is eluted from a Germanium-68/Gallium-68 generator, typically with 5 millilitres of 0.1 M hydrochloric acid, and then preheated. The [^68^Ga]GaCl_3_ solution eluted from the generator is mixed with 50 µg of the FAPI-46 precursor and heated at 90° C for 4 min (8).

Once the radiopharmaceutical has been administered intravenously, it travels in the bloodstream and enters the myocardium. Eventually, the [^68^Ga]Ga-FAPI complex binds to the fibroblast activation protein expressed on the cell membrane of activated fibroblasts. On the PET images of the myocardium, the localisation of the radiopharmaceutical in actively healing tissue will manifest as focal and sometimes diffuse increased uptake of [^68^Ga]Ga-FAPI as seen in cardiac amyloidosis. This has been demonstrated in case reports and retrospective studies conducted on patients with malignant or inflammatory diseases (9–11).

## 3. Preclinical studies supporting the use of a Gallium-68 fibroblast activation protein inhibitor post myocardial infarction

Preclinical studies have shown promising results on the role of [^68^Ga]Ga-FAPI in studying cardiac remodelling after myocardial infarction (MI) and in heart failure. Varasteh et al. induced MI in 20 rats by ligating the left anterior descending coronary artery, and a sham procedure was performed in four rats (2). A series of [^68^Ga]Ga-FAPI-04 positron emission tomography/computerised tomography (PET/CT) images were acquired on days 1, 3, 6, 14, 23, and 30 after MI, and the [^68^Ga]Ga-FAPI uptake peaked on day 6 and decreased rapidly by day 14. Immunofluorescence staining analyses on infarcted hearts on day 7 showed selective accumulation of FAP-positive cells in the peri-infarct zone (2).

Also, Qiao et al. noninvasively monitored reparative fibrosis in rats using [^68^Ga]Ga-FAPI PET/CT. In their study, they induced myocardial infarction in 16 rats by performing a thoracotomy and ligating the left anterior descending coronary artery (12). A thoracotomy was done on another 17 rats without ligating any coronary arteries (sham procedure). Serial imaging with [^68^Ga]Ga-FAPI PET/CT was performed in rats at different time points, from day 1 to day 35 post-surgery. Also, the excised cardiac tissue specimens from two rats with induced myocardial infarction and two sham-operated rats were subjected to histological examination, autoradiography, and immunofluorescence staining.

In the rats with MI, the excised cardiac tissue revealed an infiltrate of inflammatory cells, dissolved and broken muscle fibres, necrosis, and features of replacement fibrosis. Autoradiography showed an accumulation of FAPI in the infarcted myocardium and infarct border zone (12). Similarly, FAP-positive cells were identified in the infarcted area, specifically on days 3, 6, and 15 after the infarction. The myocardium of sham-operated rats exhibited normal morphology with neatly arranged myocardial fibres (12). Both of these studies demonstrated the feasibility of monitoring ventricular remodelling after MI.

Gallium-68 FAPI PET/CT findings have also been correlated with histopathological changes in rats with heart failure. Serial imaging was performed on the experimental and control groups of mice on day 0 before inducing heart failure with isoproterenol hydrochloride and 7, 14, 21, and 28 days thereafter (13). In rats with heart failure, [^68^Ga]Ga-FAPI uptake increased on days 7 and 14 and declined on days 21 and 28. Histological evaluation of heart tissue specimens showed fibrotic activity, which increased from day 0 to day 28 in rats with heart failure. No tracer uptake was seen on day 0, and a peak uptake was observed on day 7. Serial echocardiography findings showed a decline in systolic function on day 7. After day 7, ventricular chamber enlargement, ventricular wall thinning, and a reduction in myocardial contractility were observed. Also, the heart-to-muscle uptake ratio was the highest on day 7, gradually decreasing over time (13).

## 4. Clinical studies demonstrating the use of a Gallium-68 fibroblast activation protein inhibitor positron emission tomography in patients with various cardiovascular diseases

We conducted a systematic literature search on PubMed and Web of Science to identify original research studies and case reports demonstrating the use of FAPI on subjects known or suspected to have underlying cardiovascular diseases (CVD). The following search terms and associated Medical Subject Headings (MeSH) were used: “Gallium-68 AND Fibroblast activation protein” OR “Flourine-18 AND Fibroblast activation protein.” We retrieved 15 studies focused on the clinical utility of FAPI-based PET tracers labelled with either Gallium-68 or Flourine-18 on conditions such as coronary artery diseases, cardiomyopathies, infiltrative heart diseases, immune checkpoint inhibitor-related myocarditis, systemic sclerosis, and other pathologies such as pulmonary arterial hypertension, which may lead to fibrotic changes in the right ventricle (Table 1). Most of the evidence supporting the potential role of FAPI-based tracers in identifying active cardiac disease and managing CVD originates from original research studies with small sample sizes, case reports, or a retrospective review of FAPI PET images in patients with malignancies referred for PET imaging. In studies reporting incidental visualisation of cardiac uptake of FAPI in patients with underlying malignancies, logistic regression analysis reporting odds ratios and correlation studies reporting correlation coefficients were used to associate the visualisation of FAPI with CVD or its risk factors.

**Table 1:** Summary of original clinical research studies evaluating the uptake of fibrin-activating protein inhibitors in the myocardium of subjects with known or suspected cardiovascular disease.

Author (Year)	Radiopharmaceutical (Radioactivity)	Study design	Sample size	Age (years)	Clinical Indication	Imaging Protocol	Imaging Findings
Wang et al. (14)	Fluorine 18 [^18^F]-AlF-NOTA-FAPI (^18^F- FAPI) (2.5–3.0 MBq/kg)	Case vs. control	72 Hypertrophic cardiomyopathy (HCM), (*n *= 50)	Cases: 43.0 ± 13.0	To explore the characteristics of cardiac FAPI PET/CT imaging and its relationship with the risk of sudden cardiac death (SCD) in HCM.	Images were acquired 60 min post tracer injection.	Patients with HCM had intense but inhomogeneous FAPI activity in the LV, which was higher than that of control participants.
			Controls (*n *= 22)	Controls: 45.0 ± 17.0			Controls: no abnormal cardiac FAPI uptake visualised.
							Myocardial uptake of ^18^F-FAPI was associated with a 5-year sudden cardiac death risk score (*r *= 0.32, *p *= 0.03).
Wang et al. (15)	[^68^Ga]Ga-FAPI -04 (157.3 ± 25.2 MBq)	Prospective	29 Dilated cardiomyopathy (DCM) (*n* = 10), Inflammatory cardiomyopathies with connective tissue disorders (*n* = 10), Hypertrophic cardiomyopathy (HCM) (*n* = 3), Left ventricular noncompaction (LVNC) (*n* = 3), Restrictive cardiomyopathy	43.14 ± 16.94	To investigate *in vivo* myocardial fibroblast activation in different subtypes of non-ischaemic cardiomyopathies	[^68^Ga]Ga-FAPI -04 PET/CT performed 60 min after tracer administration.	Inhomogeneous increased ^68^Ga-FAPI-04 uptake in the left ventricle in 22 (75.9%) patients. Among the 22 patients, 10 (34.5%) showed slightly diffuse uptake in the right ventricle, including 1 RCM patient, 2 DCM patients, 3 HCM patients and 4 IC patients.
			(RCM) (*n* = 1), Hyperthyroidism-induced cardiomyopathy (HIC) (*n* = 1) Immune checkpoint inhibitor-related myocarditis (ICIM) (*n* = 1)			SUVR defined as the SUVmean of the myocardial volume of interest (VOI) divided by the SUVmean of the blood pool of descending thoracic aorta VOI (1 cm^3^).	Uptake of tracer seen in 22 patients: SUVmax = 4.16 ± 2.75, SUVmean = 2.09 ± 1.31, SUVR = 1.92 ± 1.18.
							Left ventricular metabolism volume (LVMV) = 196.13 ± 64.93.
							SUVmax, SUVR, and the LVMV correlated with the LVEDD (*r *= 0.407, *P *= 0.031; *r *= 0.424, *P *= 0.025; and *r *= 0.636, *P *= 0.002, respectively).
							Correlation between the LVMV and the LVESD (*r *= 0.545, *P *= 0.011).
Wang et al. (16)	[^68^Ga]Ga-FAPI -04	Prospective	30		Detection of fibroblast activation in patients with biopsy-proven systemic amyloid light chain amyloidosis		Increased left ventricular tracer uptake was visualised in 24 of 30 (80%).
							Among the 24 patients, 20 had a diffuse pattern of tracer uptake, and four had patchy uptake.
							The SUV_mean_ correlated with NT-proBNP levels (*r *= 0.625), LVESV (*r *= 0.607), and the ECV % (*r *= 0.519)
Song et al. (13)	[^68^Ga]Ga-FAPI-04 (1.8–2.2 MBq/kg) and ^13^N-NH3	Prospective	7 Heart failure patients and retrospective ^68^Ga-FAPI data from 20 subjects without cardiovascular diseases (CVD)	31–75	To assess the suitability of using [^68^Ga]Ga-FAPI PET to quantify cardiac FAP and visualise cardiac fibrosis in patients with HF secondary to DCM, HCM, and CAD.	Nitrogen-13 ammonia PET perfusion imaging followed by ^68^Ga-FAPI injection 2 h later. Imaging at 45-minute time points for 20 min.	[68Ga]Ga-FAPI uptake was inconsistent with ^13^N-NH3 perfusion. Diffuse FAPI uptake, sometimes slight. SUV_max_ normalised (2.57–9.00)
Zhang et al. (17)	[^68^Ga]Ga-DOTA-FAPI-04 (2.2 ± 0.2 MBq/kg)	Prospective	26 patients referred for percutaneous coronary intervention (PCI) for ST-elevation myocardial infarction (STEMI)	62.0 ± 8.4	To quantitatively assess the longitudinal changes in the intensity and extent of myocardial fibroblast activation and explore its predictive value for late LV remodelling approximately 12 months after acute myocardial infarction (MI).	Baseline cardiac [^68^Ga]Ga-DOTA-FAPI-04 PET/MR scans done after a mean duration of 4.5 ± 1.5 days (3–8 days) after STEMI.	Correlation between [^68^Ga]Ga-DOTA-FAPI-04 uptake volume (UV) and the LVEDV (*r *= 0.680, *p *< 0.001), LVESV (*r *= 0.720, *p *< 0.001) and the LVEF (*r *= −0.681, *p *< 0.001) at baseline.
							The intensity and volume of [68Ga]Ga-DOTA-FAPI-04 uptake decreased from baseline to 12-month follow-up, but myocardial [^68^Ga]Ga-DOTA-FAPI-04 uptake persisted for 12 months after acute MI in all patients.
							[^68^Ga]Ga-DOTA-FAPI-04 UV was associated with an increase in the LVEDV (*r *= 0.445, *p *= 0.033) and the LVESV (*r *= 0.456, *p *= 0.029) and a decrease in the LVEF (*r *= −0.423, *p *= 0.044) over 12 months.
							Negative correlation between [^68^Ga]Ga-DOTA-FAPI-04 UV (*r *= −0.783, *p *< 0.001) and TBRmax (*r *= −0.484, *p *= 0.019) and the LVEF at the time of 12-month follow-up.
Diekmann et al. (18)	[^68^Ga]Ga-FAPI-46 (114 ± 22 MBq)	Retrospective	35 patients, after percutaneous coronary intervention (PCI) for ST-elevation myocardial infarction (STEMI)	57 ± 11	To test the hypotheses that: [^68^Ga]Ga-FAPI-46 PET reflects a myocardial signal early after acute MI that is not identical to CMR-derived tissue characteristics.	Perfusion imaging with 388 ± 32 MBq of ^99m^Tc-tetrofosmin single photon emission computed tomography (SPECT), 5.0 ± 1.5 days after acute MI.	Increased uptake of [^68^Ga]Ga-FAPI-46 PET in the territory of the culprit infarct vessel (SUVpeak, 6.4 ± 1.5) in all patients.
					[^68^Ga]Ga-FAPI-46 predicts the later development of ventricular dysfunction.	FAP-targeted PET was performed at 7.5 ± 1.3 days. PET images were acquired 60 min after tracer injection for 20 min.	Seven patients with complete reperfusion and no perfusion defects on SPECT also showed increased tracer uptake in the affected vascular territory.
						CMR: T1 and T2 weighted images, LGE	FAPI volume correlated with the maximum creatine kinase (*r *= 0.42, *p *= 0.012) and inflammatory markers (maximum C-reactive protein: *r *= 0.43, *p *= 0.010; maximum white blood cell count: *r *= 0.31, *p *= 0.07).
							Patients with diabetes mellitus had a larger FAP volume (134 ± 53 cm^3^ vs. 93 ± 36 cm^3^, *p *= 0.012).
							Correlation between the FAP volume and LV mass (*r *= 0.69, *p *= 0.001), end-diastolic volume (*r *= 0.57, *p *= 0.001), end-systolic volume (*r *= 0.62, *p *= 0.001), and LGE volume (*r *= 0.58, *p *= 0.001).
Treutlein et al. (19)	[^68^Ga]Ga-FAPI-04 (1.5 MBq/kg body weight)	Prospective	Systemic sclerosis (SSc) + myocardial fibrosis (MF), *n *= 6	SSc + MF: Median 59.5 (IQR: 58.0–63.3)	To test the hypothesis that [^68^Ga]Ga-FAPI-04 uptake can differentiate systemic sclerosis (SSc) patients with myocardial fibrosis from SSc patients without myocardial fibrosis.	[^68^Ga]Ga-FAPI-04-PET/CT, with a non-enhanced CT of the thorax after 15 min.	Increased [^68^Ga]Ga-FAPI-04 uptake in patients with SSc without myocardial fibrosis
			SSc and no MF, *n *= 6	SSc and no MF: median age 56.5 years (IQR: 47.8–67)	To test the hypothesis that increased [^68^Ga]Ga-FAPI-04 uptake is associated with unfavourable prognostic factors in SSc-MF and that [^68^Ga]Ga-FAPI-04 uptake assesses current molecular fibroblast activity rather than accumulating disease damage.		
			SSc with previous myocardial disease and no MF, *n *= 2	Controls: median age 51 years (IQR: 44.3–54.3)			
			Controls:(*n *= 2) heart-transplanted patients with healthy donor hearts				
Gu et al. (20)	[^68^Ga]Ga-FAPI-04 (1.48–1.85 MBq/kg)	Pilot study	Pulmonary arterial hypertension (PAH), (*n *= 16): PAH associated with congenital heart disease (*n *= 12)	32 ± 9 years	To evaluate the feasibility of [^68^Ga]Ga-FAPI PET imaging in assessing fibrotic remodelling in the right ventricle (RV)	The PET/CT images were acquired 20 min after tracer injection	Twelve of the 16 patients (75%) with PAH showed heterogeneous FAPI uptake in the RV-free wall and insertion point.
			Idiopathic PAH (*n *= 4)		To assess the relationship between FAPI uptake and parameters of pulmonary hemodynamics and cardiac function in pulmonary arterial hypertension (PAH)		Increased FAPI uptake in the RV-free wall (SUVmax: 2.5 ± 1.8, *P* < 0.001) and insertion point (SUVmax: 2.5 ± 1.7, *P* < 0.001)
							Normal RV function was seen in four patients without FAPI uptake.
							Patients with tricuspid annular plane systolic excursion (TAPSE) < 17 mm presented with higher FAPI uptake compared to those with TAPSE ≥ 17 mm in both the RV-free wall (SUVmax: 3.4 ± 1.9 vs. 1.7 ± 1.1, *p* = 0.010) and the insertion point (SUVmax: 3.4 ± 1.9 vs. 1.6 ± 0.7, *p* = 0.028)
							FAPI intensity correlated with total pulmonary resistance (RV-free wall: *r* = 0.529, *p* = 0.035; insertion point: *r* = 0.576, *p *= 0.02) and the level of N-terminal pro-b-type natriuretic peptide (*N*T-proBNP) (RV free wall: *r* = 0.606, *p *= 0.013; insertion point: *r *= 0.653, *p *= 0.006).
							FAPI uptake did not correlate with other pulmonary hemodynamic parameters.
Guo and Chen (21)	[^68^Ga]Ga-FAPI-46	Case report	1	66	Staging of multiple myeloma		The thickened left ventricle of the myocardium and tongue exhibited diffuse and inhomogeneous[^68^Ga]Ga-FAPI-46 uptake
					Female with a 3-month history of progressive dyspnoea		CMR: left ventricle thickening and global subendocardial LGE.
							A tongue biopsy revealed positive congo red staining, consistent with amyloid involvement.
Notohamiprodjo et al. (10)	[^68^Ga]Ga-FAPI-04 (165 MBq)	case vs. control	5 (case: *n *= 1, controls: *n *= 4)	33 (case)	CASE: Previous ST-elevation MI with persistent dyspnoea and fatigue post PCI in the LAD artery. Compassionate use for chimeric antigen receptor T-cell therapy of myocardial fibrosis and clarifying inflammation and viability after MI.	Gallium-68 FAPI-04- PET/MR (Day 6 post STEMI): Dynamic PET done.	PET: Increased and intense focal uptake in the anterior and anterior-septal walls of the myocardium.
				37–61 (controls)	CONTROLS: Staging and possible compassionate use of ^177^Lu-FAPI radiotherapy in metastatic osteosarcoma, breast cancer, tongue carcinoma, and oropharynx carcinoma	Cardiovascular magnetic resonance (CMR) T2 weighted imaging, early and late gadolinium enhancement. Cardiovascular magnetic resonance imaging was repeated six months after MI.	CMR: a small scar in the apex with no tracer uptake in the scar region.
							EGE: transmural enhancement of the anterior wall and adjacent septal segments.
							LGE: sub-endocardial enhancement in the anterior-septal and inferior aspects of the apex.
							Tracer uptake was more extensive than pathological CMR findings.
Xie et al. (22)	Fluorine 18 [^18^F]-AlF-NOTA-FAPI (^18^F- FAPI) (2.5–3.0 MBq/kg)	Prospective	14 STEMI patients subjected to primary PCI	STEMI: 62 ± 11 years	To assess the correlation between FAPI and CMR imaging parameters in reperfused STEMI patients in the acute phase	The PET/CT images were acquired 60 min after tracer injection.	All STEMI patients had localised and inhomogeneous FAPI uptake.
			14 healthy controls	Controls: 50 ± 14 years	To investigate the prognostic value of FAPI imaging in cardiac recovery three months post-MI.		No uptake was detected in controls.
					To evaluate the correlation between FAPI activity and circulating FAP and inflammatory biomarkers.		Higher tissue-to-background ratio (TBRmax) in the infarct region than in the remote area in STEMI patients (9.68 ± 2.61 vs. 1.07 ± 0.25, *p *< 0.001).
							Higher TBRmax in STEMI vs. controls (0.96 ± 0.20, *p *< 0.001).
							FAPI% larger than T2WI%.
							Correlation between FAPI% and creatine kinase-MB (CKMBmax), white blood cell count (WBCmax), and lactate dehydrogenase (LDHmax) (*r* values of 0.79, 0.65, and 0.62; all *p *< 0.05).
							Correlation between FAPI% × TBRmax and CKMBmax, WBCmax, LDHmax, and BNPmax (*r* values of 0.56, 0.55, 0.56, and 0.59; all *p *< 0.05).TBRmax was only related to WBCmax (*r *= 0.59, *p *= 0.03).
Kessler et al. (23)	[^68^Ga]Ga-FAPI-46 (142.8 ± 27.5 )	Retrospective	10	63.6 ± 12.5	Risk stratification post MI and PCI	Dynamic imaging for 20 min, whole-body PET scan at 60 min post tracer injection for 10 min.	Ten patients had moderate-to-intense tracer uptake.
						Fibroblast activation volume (FAV) was measured using the 40% volume-of-interest isocontour.	Activated fibroblasts inversely correlated with the LVEF (*r *= −0.69, *p* < 0.05).
							Activated fibroblasts correlated with the maximum CK (*r *= 0.90, *p* < 0.01), reflecting the extent of myocardial damage. SUV_max_ of 8.9 ± 4.4 (range, 5.5–17.4), SUV_peak_ of 7.6 ± 4.0, and an SUV_mean_ of 5.3 ± 2.8 (10 min after tracer administration)
Finke et al. (24)	[^68^Ga]Ga-FAPI (122–336 MBq)	Prospective	26 no cardiac disease, (*n *= 23), suspected immune checkpoint inhibitors (ICI)-associated myocarditis, (*n *= 3)	62–74	Diagnosis of ICI-associated myocarditis in cancer patients previously treated with ICI.	PET imaging 60 min post tracer injection	Tracer uptake in the neoplastic tissues visualised.
							Three patients with biopsy-proven auto-immune myocarditis: Patient 1:Diffuse tracer uptake in the left ventricle.
							Patient 2: Localised uptake in the septum.
							Patient 3: Localised uptake in the apical posterior wall of the left ventricle.
							Controls: No tracer uptake
							Myocarditis patients: the median SUV was 1.79 (IQR: 1.65–1.85)
							Non-myocarditis patients: median SUV 1.15 (IQR: 0.955–1.52).
Siebermair et al. (25)	[^68^Ga]Ga-FAPI -04 (140 ± 24 MBq)	Retrospective review	32	58.7 ± 14.9	To assess patterns of myocardial uptake of [^68^Ga]Ga-FAPI in patients with malignancies	PET imaging 12 ± 7 min post tracer injection.	Focal tracer accumulation was noted in six patients (19%). Univariate regression showed a weak but significant correlation between SUV_mean_ and CAD (*r*^2 ^= 0.16, *p *= 0.03), MI (*r*^2 ^= 0.14, *p *= 0.04) and age (*r*^2 ^= 0.15, *p *= 0.04). SUVmax 7.1 ± 4.8, *p* < 0.05, SUVmean 5.2 ± 4.0, *p* < 0.05.
Heckmann et al. (26)	[^68^Ga]Ga-FAPI (122–336 MBq)	Retrospective	229 (Initial cohort: =185, confirmatory cohort: =44)	64−77	To evaluate cardiac tracer accumulation and its correlation with CVD in patients with malignancies referred for imaging with [^68^Ga]Ga-FAPI PET/CT	PET imaging 60 min post tracer injection, while some patients were also imaged at 10 and 180 min after tracer injection	Five patterns of tracer uptake: homogenous, diffuse, focal on diffuse, focal, and weak enrichment.
							A focal pattern of tracer uptake was seen in more patients with cardiovascular risk factors (*p* < 0.0001, Yate *χ*2-test).
							Increased uptake of tracer associated with thyroid stimulating hormone (TSH) serum levels > 4 µU/ml (OR = 8.6, *p *= 0.012), BMI > 25 kg/m^2^ (OR = 2.6, *p *= 0.023), and diabetes mellitus (OR = 2.9, *p *= 0.041)
							Previous chest radiation (OR = 3.5, *p *= 0.024) was associated with a higher FAP signal on logistic regression analysis.
							Focal tracer uptake was associated with cardiovascular risk factors, CAD, and oral aspirin intake.

BMI, body mass index; CAD, coronary artery disease; CK, creatine kinase; CMR, cardiovascular magnetic resonance; CVD, cardiovascular disease; CT, computed tomography; DCM, dilated cardiomyopathy; EGE, early gadolinium enhancement; FAPI, fibroblast activation protein inhibitor; FAV, fibroblast activation volume; HCM, hypertrophic cardiomyopathy; HF, heart failure; ICI, immune checkpoint inhibitors; IQR, interquartile range; Kg, kilogram; LAD, left anterior descending; LGE, late gadolinium enhancement; LV, left ventricle; LVEDD, left ventricular end-diastolic diameter; LVEF, left ventricular ejection fraction; LVMV, left ventricular metabolic volume; LVESD, left ventricular end systolic diameter; LVESV, left ventricular end systolic volume; ^177^Lu, lutetium-177; MBq, megabecquerel; MI, myocardial infarction; ^13^N-NH3, nitrogen-13 ammonia; NT-proBNP, N-terminal pro-brain natriuretic peptide; OR, odds ratio; PCI, percutaneous coronary intervention; PET, positron emission tomography; RV, right ventricle; SSc, systemic sclerosis; STEMI, ST-elevation myocardial infarction; SUV, standardized uptake value; TAPSE, tricuspid annular plane systolic excursion; TSH, thyroid stimulating hormone; VOI, volume of interest.

### 4.1. Coronary artery disease

FAPI for ST-segment elevation myocardial infarction (STEMI) has been primarily used in patients with coronary artery disease. Zhang et al. studied 26 patients with STEMI after percutaneous coronary intervention referred for imaging with [^68^Ga]Ga-DOTA-FAPI Positron emission tomography/magnetic resonance imaging (PET/MRI) and found that both the volume and intensity of FAPI decreased over time when comparing the baseline and follow-up scan performed 12 months later. However, on the PET/MR images acquired 12 months after the acute myocardial infarction, FAPI uptake persisted in all patients studied (17). Similarly, in another study involving 35 patients with STEMI, [^68^Ga]Ga-DOTA-FAPI uptake was significantly elevated in the territory of the stenosed coronary artery (18).

Atherosclerotic coronary artery disease, presenting as chronic coronary or acute unstable syndromes, results from traditional risk factors such as hypertension and smoking, known to cause coronary vascular endothelial damage by inducing inflammation, as evidenced by elevated C-reactive protein plasma levels (27). In addition, atherosclerosis may occur as a secondary element of vascular inflammation, despite the absence of cardiovascular risk factors (28, 29). Acute coronary syndromes are precipitated by the thrombotic occlusion of an unstable, complicated atherosclerotic plaque. During myocardial ischaemia, the reduction in blood flow and subsequent delivery of oxygen to the heart muscle induces necrosis in the myocyte. Once the vessel is damaged, it attempts to “seal” the damaged area by depositing lipids and recruiting inflammatory cells (30). The diameter of the vessel where the atheroma is located narrows over time.

In response to myocardial ischaemia, a series of signalling mechanisms lead to the transformation of the structure of fibroblasts from the resting state to an activated proto-myofibroblast, which ultimately transforms into a myofibroblast (Figure 1).

Recurrent, small thrombotic non-occlusive ischaemic episodes and reperfusion or occlusive non-reperfused episodes are usually followed by the recruitment of macrophages and fibroblasts to the injured area of the myocardium or endocardium, a hallmark of myocardial fibrosis. As demonstrated by Zhang and colleagues, the persistent activation of fibroblasts may suggest the presence of underlying ventricular remodelling, in which the heart attempts to repair the infarcted area, or adverse ventricular remodelling, where the recruitment of fibroblasts will result in excessive deposition of collagen, which will eventually impair the contractility of the heart muscles.

**Figure 2:**
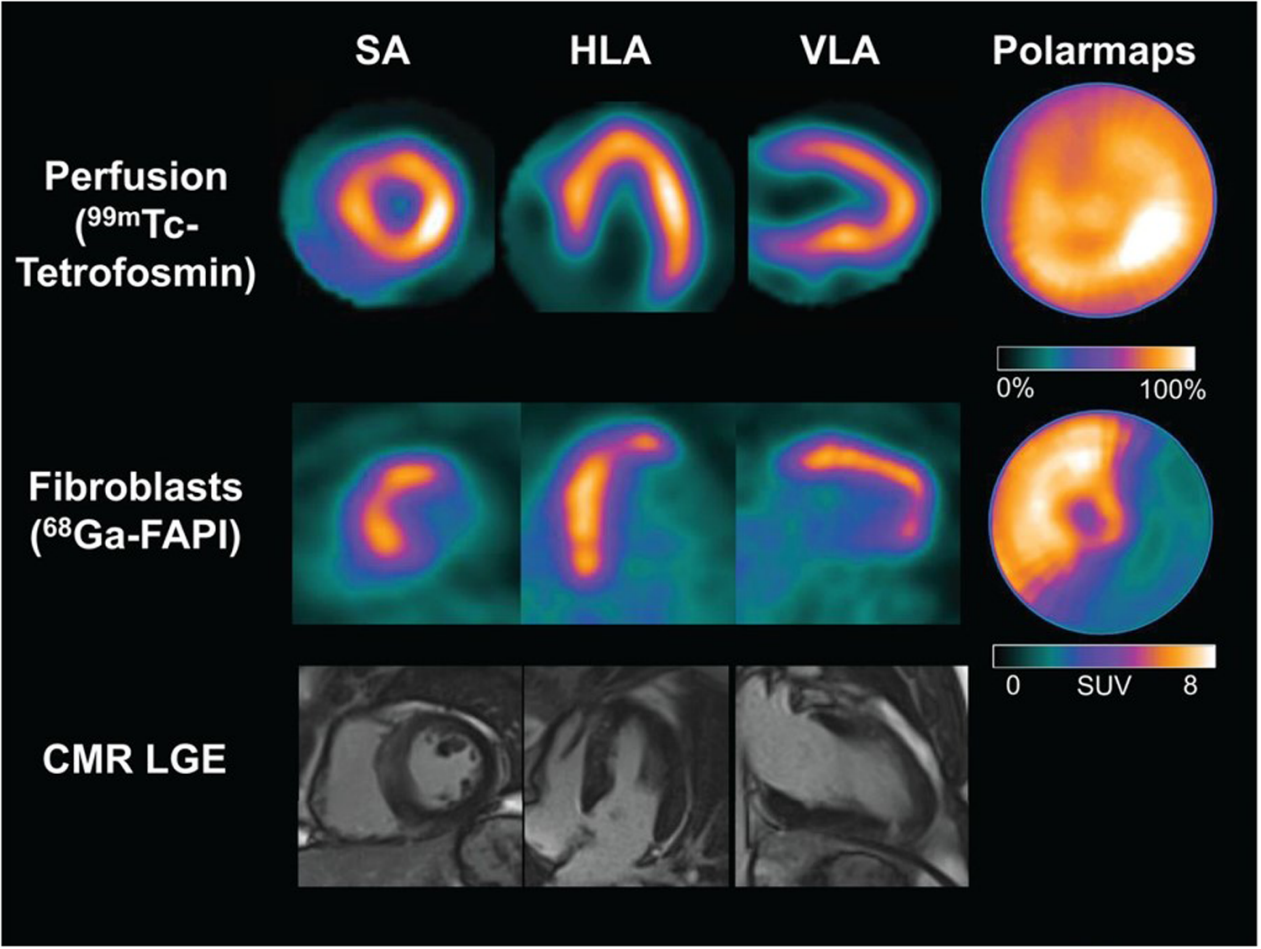
Cardiac images acquired after reperfusion therapy for acute myocardial infarction. The first row depicts single photon emission computed tomography (SPECT) myocardial perfusion images acquired at rest using [^99m^Tc]Tc-tetrofosmin, followed by [^68^Ga]Ga-FAPI-04 PET images in the second row. The third row depicts cardiovascular magnetic resonance imaging. There are no perfusion defects visualised after reperfusion therapy in the SPECT resting perfusion images. However, a large area of cardiac fibroblast activation is visualised in the anterior and septal walls and partially in the apex. There is no evidence of late gadolinium enhancement in the corresponding myocardial images on CMR. HLA, horizontal long axis; SA, short axis; VLA, vertical long axis. This research was originally published in *JNM*. Johanna Diekmann et al. (18). © SNMMI.

Non-invasive fibroblast activation detection after acute myocardial infarction may identify areas of adverse ventricular remodelling depicted by persistent [^68^Ga]Ga-FAPI-04 uptake despite re-perfusion therapy. This persistent uptake has been demonstrated by Diekmann et al., who studied 35 patients with post acute STEMI. These patients were referred for imaging with single photon emission computed tomography (SPECT), PET, and CMR after percutaneous coronary intervention and dual-antiplatelet therapy (18). Despite receiving reperfusion therapy, [^68^Ga]Ga-FAPI-04 PET images showed uptake in the anterior and septal walls and, partially, in the apex (Figure 2)(18). The clinical significance of cardiac fibroblast activation after reperfusion therapy needs further exploration, as this finding may be indicative of adverse or expected ventricular remodelling after restoring myocardial perfusion.

### 4.2. Hypertrophic cardiomyopathy

Fibroblast activation has been studied in patients with dilated and hypertrophic cardiomyopathies (14, 15). In patients with hypertrophic cardiomyopathy (HCM), interstitial fibrosis is one of the typical histological features predisposing patients to arrhythmias and heart failure (31). Wang et al. performed PET imaging with Fluorine 18 [^18^F]-AlF-NOTA-FAPI (^18^F-FAPI) on 50 patients with HCM and 22 age and sex-matched healthy volunteers (14). They found intense and inhomogeneous tracer uptake in all patients with HCM, and the uptake was weakly correlated (*r* = 0.32) with a 5-year risk of sudden cardiac death (14). The potential role of FAPI-based tracers in HCM is for the selection of patients at high risk of sudden cardiac death (SCD), where an implantable cardioverter-defibrillator (ICD) may be implanted prophylactically to prevent lethal arrhythmias and heart failure in patients exhibiting FAPI uptake on imaging.

### 4.3. Amyloidosis

Amyloidosis is a systemic infiltrative disease characterised by the extracellular deposition of insoluble proteins in various organs, including the heart, and the abnormal accumulation of amyloid proteins in solid organs may lead to organ dysfunction (32). Non-invasive imaging modalities such as echocardiography and CMR imaging help to identify cardiac involvement in subjects with systemic amyloidosis (32). In a study involving 30 patients with biopsy-proven systemic light-chain amyloidosis, [^68^Ga]Ga-FAPI-04 PET/CT was used to assess cardiac fibroblast activation (16). Patchy and diffuse uptake patterns were found in 80% of patients, suggesting active cardiac remodelling (16). Gou and Chen explored the clinical utility of staging multiple myeloma with [^68^Ga]Ga-FAPI-04 PET/CT. Diffuse and inhomogeneous FAPI uptake was visualised in the left ventricle, suggesting cardiac amyloidosis (21). In patients with cardiac involvement, FAPI-based imaging may also play a role in identifying patients at risk for SCD requiring ICDs.

## 5. Assessment of myocardial fibrosis

Fibrosis in the heart indicates an area in the myocardium that cannot contract effectively, leading to myocardial contractile dysfunction, heart failure, a possible nidus for ventricular arrhythmias, and SCD (33–35). The endomyocardial biopsy allows for histological, immunohistochemical, and molecular evaluation of a specimen of cardiac tissue (36). Ideally, the biopsy should be performed under image guidance to increase the probability of sampling abnormal tissue. The major drawback of performing an endomyocardial biopsy is the limited access to cardiac catheterisation laboratories in most low-and middle-income countries.

Newer imaging techniques, such as the visualisation of late gadolinium enhancement and the quantification of the extracellular volume using CMR imaging, have proven to be helpful in identifying fibrotic tissue in patients with ischaemic and non-ischaemic dilated cardiomyopathy (37–39). In research settings, human cardiac tissue has been excised during coronary artery bypass graft (CABG) surgery, left ventricular assist device implantation, and cardiac transplant surgery to assess for micro- and macroscopic evidence of myocardial fibrosis (40). The characteristics of non-invasive imaging methods for evaluating myocardial fibrosis are summarised in Table 2.

**Table 2:** Non-invasive imaging modalities for the assessment of myocardial fibrosis.

	Technique	Advantages	Limitations
Positron Emission Tomography with [^68^Ga]Ga-FAPI (41)	[^68^Ga]Ga-FAPI binds the fibroblast activation protein expressed on the transmembrane surface of activated fibroblasts. This technique images sites of active remodelling in the heart and may not directly serve as an imaging marker of established fibrosis.	– Possible theranostic applications – Detection of possible pre-fibrotic disease – Detection of early manifestations of cardiac remodelling.	– Cost – Exposure to ionising radiation – Randomised control trials or outcome-based studies are still lacking
	Imaging finding: Focal or diffuse accumulation of tracer in sites of active remodelling.		
Positron Emission Tomography with 2-Deoxy-2-[18F]fluoro-d-glucose [2-(^18^F)FDG] (42)	2-[^18^F]FDG, which is transported *via* glucose transporters into cardiac myocytes, confirms the presence of metabolic activity in hibernating or viable tissue by localising in segments of the myocardium with reduced perfusion and contractile dysfunction.	– High sensitivity for detecting viable myocardium – Selects patients whose ventricular function might improve after successful coronary revascularisation	– Exposure to ionising radiation – Cost – Patient preparation significantly impacts image interpretation
	Scar or fibrotic tissue on imaging manifests as absent perfusion and decreased or absent metabolic activity (perfusion-metabolism match)		
Single Photon Emission Computed Tomography (43)	Preserved or slightly reduced perfusion, normal thickening, and preserved wall motion may suggest the presence of viable, non-fibrotic myocardial tissue	– Further metabolic imaging is required to differentiate between fibrosis and hibernating tissue in segments with ≥50% reduction in perfusion and abnormal wall motion. – Useful for risk stratification and predicting outcomes such as mortality – Estimate ventricular volume and function	– Ionising radiation
Cardiovascular Magnetic Resonance Imaging (44)	T1 mapping tracks the recovery of longitudinal magnetisation	– Useful for risk stratification and predicting outcomes such as mortality – Volumetric quantification – High specificity	– Lack of standardisation in data acquisition and post-processing
	Late gadolinium enhancement: gadolinium retention in the expanded extracellular space after loss of myocytes. Retained gadolinium leads to the enhancement of fibrotic tissue in the images.		
	Extracellular volume mapping: expansion of the extracellular volume space		
Echocardiography (45)	Speckle tracking measures the extent of the myocardial deformity using parameters such as global longitudinal and circumferential strain. Global longitudinal strain correlates with myocardial fibrosis in advanced systolic heart failure.	– Portable and readily accessible	– Operator dependent – Image quality influenced by body habitus
Computerised tomography (44)	Late iodine enhancement: delayed clearance of iodinated contrast media	– Excellent spatial resolution – High temporal resolution	– Low signal-to-noise ratio
** **	Extracellular volume mapping: expansion of the extracellular volume space secondary to interstitial fibrosis		

## 6. Future Studies and Recommendations

Cardiac fibroblast activation has been studied in patients with various CVDs, mostly in patients with ischaemic heart disease. Whether the visualisation of FAPI uptake on the myocardium suggests the presence of an underlying normal response to myocardial injury or adverse remodelling remains to be elucidated. Future studies should attempt to perform non-invasive imaging at multiple time points and correlate imaging findings with inflammatory markers. The frequency of imaging could be extrapolated from animal models. In addition, subjects known to have coronary artery diseases should be subjected to coronary angiography and subsequently randomised to imaging with FAPI-based tracers and CMR imaging to evaluate the clinical impact of FAPI in defining clinically relevant outcomes such as cardiovascular death, all-cause mortality, and the rate of rehospitalisation. Furthermore, considering that the activation of fibroblasts is a momentary phase in the life cycle of fibroblasts, the window of opportunity for intervening should be clearly defined. Moreover, the utility of FAPI-based tracers could be further explored in patients with chronic coronary syndromes, potentially selecting candidates for coronary revascularisation.

## 7. Conclusions

Evidence supporting the application of FAPI-based radiopharmaceuticals in cardiac diseases is still in its infancy, comprising data collated from original research studies with small sample sizes, case reports, and retrospective studies on patients with oncological conditions. After a myocardial injury, the heart attempts to repair the damaged tissue and maintain its structural integrity by orchestrating a series of processes, including the deposition of extracellular matrix components and the activation of fibroblasts. Unresolved pertinent issues related to imaging activated fibroblasts include the appropriate timing for imaging and the need for a definitive management plan in patients with FAPI uptake in the myocardium.

## References

[1] Kalluri R. The biology and function of fibroblasts in cancer. Nat Rev Cancer. (2016) 16(9):582–98. 10.1038/nrc.2016.73 27550820

[2] Varasteh Z Mohanta S Robu S Braeuer M Li Y Omidvari N Molecular imaging of fibroblast activity after myocardial infarction using a (68)Ga-labeled fibroblast activation protein inhibitor, FAPI-04. J Nucl Med. (2019) 60(12):1743–9. 10.2967/jnumed.119.226993 31405922 PMC6894377

[3] Lindner T Loktev A Giesel F Kratochwil C Altmann A Haberkorn U. Targeting of activated fibroblasts for imaging and therapy. EJNMMI Radiopharm Chem. (2019) 4(1):16. 10.1186/s41181-019-0069-0 31659499 PMC6658625

[4] Sollini M Kirienko M Gelardi F Fiz F Gozzi N Chiti A. State-of-the-art of FAPI-PET imaging: a systematic review and meta-analysis. Eur J Nucl Med Mol Imaging. (2021) 48(13):4396–414. 10.1007/s00259-021-05475-0 34173007

[5] Liermann J Syed M Ben-Josef E Schubert K Schlampp I Sprengel SD Impact of FAPI-PET/CT on target volume definition in radiation therapy of locally recurrent pancreatic cancer. Cancers (Basel). (2021) 13(4):1–13. 10.3390/cancers13040796 PMC7918160 33672893

[6] Lindner T Loktev A Altmann A Giesel F Kratochwil C Debus J Development of quinoline-based theranostic ligands for the targeting of fibroblast activation protein. J Nucl Med. (2018) 59(9):1415–22. 10.2967/jnumed.118.210443 29626119

[7] Huang R Pu Y Huang S Yang C Yang F Pu Y FAPI-PET/CT in cancer imaging: a potential novel molecule of the century. Front Oncol. (2022) 12:854658. 10.3389/fonc.2022.854658 35692767 PMC9174525

[8] Alfteimi A Lutzen U Helm A Juptner M Marx M Zhao Y Automated synthesis of [(68)Ga]Ga-FAPI-46 without pre-purification of the generator eluate on three common synthesis modules and two generator types. EJNMMI Radiopharm Chem. (2022) 7(1):20. 10.1186/s41181-022-00172-1 35904684 PMC9338183

[9] Lyu Z Han W Zhao H Jiao Y Xu P Wang Y A clinical study on relationship between visualization of cardiac fibroblast activation protein activity by Al(18)F-NOTA-FAPI-04 positron emission tomography and cardiovascular disease. Front Cardiovasc Med. (2022) 9:921724. 10.3389/fcvm.2022.921724 36072860 PMC9441604

[10] Notohamiprodjo S Nekolla SG Robu S Villagran Asiares A Kupatt C Ibrahim T Imaging of cardiac fibroblast activation in a patient after acute myocardial infarction using (68)Ga-FAPI-04. J Nucl Cardiol. (2022) 29(5):2254–61. 10.1007/s12350-021-02603-z 33860458 PMC9553764

[11] Windisch P Zwahlen DR Giesel FL Scholz E Lugenbiel P Debus J Clinical results of fibroblast activation protein (FAP) specific PET for non-malignant indications: systematic review. EJNMMI Res. (2021) 11(1):18. 10.1186/s13550-021-00761-2 33606104 PMC7895887

[12] Qiao P Wang Y Zhu K Zheng D Song Y Jiang D Noninvasive monitoring of reparative fibrosis after myocardial infarction in rats using (68)Ga-FAPI-04 PET/CT. Mol Pharm. (2022) 19(11):4171–8. 10.1021/acs.molpharmaceut.2c00551 35969029

[13] Song W Zhang X He S Gai Y Qin C Hu F (68)Ga-FAPI PET visualize heart failure: from mechanism to clinic. Eur J Nucl Med Mol Imaging. (2022) 50:475–85. 10.1007/s00259-022-05994-4 36269382

[14] Wang L Wang Y Wang J Xiao M Xi XY Chen BX Myocardial activity at (18)F-FAPI PET/CT and risk for sudden cardiac death in hypertrophic cardiomyopathy. Radiology. (2023) 306(2):e221052. 10.1148/radiol.221052 36219116

[15] Wang J Huo L Lin X Fang L Hacker M Niu N Molecular imaging of fibroblast activation in multiple non-ischemic cardiomyopathies. EJNMMI Res. (2023) 13(1):39. 10.1186/s13550-023-00986-3 37155002 PMC10167070

[16] Wang X Guo Y Gao Y Ren C Huang Z Liu B Feasibility of (68)Ga-labeled fibroblast activation protein inhibitor PET/CT in light-chain cardiac amyloidosis. JACC Cardiovasc Imaging. (2022) 15(11):1960–70. 10.1016/j.jcmg.2022.06.004 36357138

[17] Zhang M Quan W Zhu T Feng S Huang X Meng H [(68)Ga]ga-DOTA-FAPI-04 PET/MR in patients with acute myocardial infarction: potential role of predicting left ventricular remodeling. Eur J Nucl Med Mol Imaging. (2023) 50(3):839–48. 10.1007/s00259-022-06015-0 36326870 PMC9852131

[18] Diekmann J Koenig T Thackeray JT Derlin T Czerner C Neuser J Cardiac fibroblast activation in patients early after acute myocardial infarction: integration with MR tissue characterization and subsequent functional outcome. J Nucl Med. (2022) 63(9):1415–23. 10.2967/jnumed.121.263555 35210301 PMC9454470

[19] Treutlein C Distler JHW Tascilar K Fakhouri SC Gyorfi AH Atzinger A Assessment of myocardial fibrosis in patients with systemic sclerosis using [(68)Ga]ga-FAPI-04-PET-CT. Eur J Nucl Med Mol Imaging. (2023) 50(6):1629–35. 10.1007/s00259-022-06081-4 36522438 PMC10119041

[20] Gu Y Han K Zhang Z Zhao Z Yan C Wang L (68)Ga-FAPI PET/CT for molecular assessment of fibroblast activation in right heart in pulmonary arterial hypertension: a single-center, pilot study. J Nucl Cardiol. (2023) 30(2):495–503. 10.1007/s12350-022-02952-3 35322381

[21] Guo W Chen H. (68)Ga FAPI PET/MRI in cardiac amyloidosis. Radiology. (2022) 303(1):51. 10.1148/radiol.211951 34931860

[22] Xie B Wang J Xi XY Guo X Chen BX Li L Fibroblast activation protein imaging in reperfused ST-elevation myocardial infarction: comparison with cardiac magnetic resonance imaging. Eur J Nucl Med Mol Imaging. (2022) 49(8):2786–97. 10.1007/s00259-021-05674-9 34984503

[23] Kessler L Kupusovic J Ferdinandus J Hirmas N Umutlu L Zarrad F Visualization of fibroblast activation after myocardial infarction using 68Ga-FAPI PET. Clin Nucl Med. (2021) 46(10):807–13. 10.1097/RLU.0000000000003745 34477601

[24] Finke D Heckmann MB Herpel E Katus HA Haberkorn U Leuschner F Early detection of checkpoint inhibitor-associated myocarditis using (68)Ga-FAPI PET/CT. Front Cardiovasc Med. (2021) 8:614997. 10.3389/fcvm.2021.614997 33718446 PMC7946849

[25] Siebermair J Köhler MI Kupusovic J Nekolla SG Kessler L Ferdinandus J Cardiac fibroblast activation detected by ga-68 FAPI PET imaging as a potential novel biomarker of cardiac injury/remodeling. J Nucl Cardiol. (2021) 28(3):812–21. 10.1007/s12350-020-02307-w 32975729 PMC8249249

[26] Heckmann MB Reinhardt F Finke D Katus HA Haberkorn U Leuschner F Relationship between cardiac fibroblast activation protein activity by positron emission tomography and cardiovascular disease. Circ Cardiovasc Imaging. (2020) 13(9):e010628. 10.1161/circimaging.120.010628 32912030 PMC7497888

[27] Sesso HD Buring JE Rifai N Blake GJ Gaziano JM Ridker PM. C-reactive protein and the risk of developing hypertension. JAMA. (2003) 290(22):2945–51. 10.1001/jama.290.22.2945 14665655

[28] Avina-Zubieta JA Choi HK Sadatsafavi M Etminan M Esdaile JM Lacaille D. Risk of cardiovascular mortality in patients with rheumatoid arthritis: a meta-analysis of observational studies. Arthritis Rheum. (2008) 59(12):1690–7. 10.1002/art.24092 19035419

[29] Tsabedze N Seboka M Mpanya D Solomon A. Extensive triple vessel coronary artery disease in a young male with juvenile idiopathic arthritis. Oxf Med Case Rep. 2021;2021(11-12):omab119. 10.1093/omcr/omab119 PMC8713581 34987849

[30] Falk E. Pathogenesis of atherosclerosis. J Am Coll Cardiol. 2006;47(8 Suppl):C7–12. 10.1016/j.jacc.2005.09.068 16631513

[31] Marian AJ Braunwald E. Hypertrophic cardiomyopathy: genetics, pathogenesis, clinical manifestations, diagnosis, and therapy. Circ Res. (2017) 121(7):749–70. 10.1161/CIRCRESAHA.117.311059 28912181 PMC5654557

[32] Muchtar E Dispenzieri A Magen H Grogan M Mauermann M McPhail ED Systemic amyloidosis from A (AA) to T (ATTR): a review. J Intern Med. (2021) 289(3):268–92. 10.1111/joim.13169 32929754

[33] Disertori M Rigoni M Pace N Casolo G Mase M Gonzini L Myocardial fibrosis assessment by LGE is a powerful predictor of ventricular tachyarrhythmias in ischemic and nonischemic LV dysfunction: a meta-analysis. JACC Cardiovasc Imaging. (2016) 9(9):1046–55. 10.1016/j.jcmg.2016.01.033 27450871

[34] Ekstrom K Lehtonen J Kandolin R Raisanen-Sokolowski A Salmenkivi K Kupari M. Incidence, risk factors, and outcome of life-threatening ventricular arrhythmias in giant cell myocarditis. Circ Arrhythm Electrophysiol. (2016) 9(12):1–8. 10.1161/CIRCEP.116.004559 27913400

[35] Mc LA Ellims AH Prabhu S Voskoboinik A Iles LM Hare JL Diffuse ventricular fibrosis on cardiac magnetic resonance imaging associates with ventricular tachycardia in patients with hypertrophic cardiomyopathy. J Cardiovasc Electrophysiol. (2016) 27(5):571–80. 10.1111/jce.12948 26840595

[36] Porcari A Baggio C Fabris E Merlo M Bussani R Perkan A Endomyocardial biopsy in the clinical context: current indications and challenging scenarios. Heart Fail Rev. (2023) 28(1):123–35. 10.1007/s10741-022-10247-5 35567705 PMC9107004

[37] Memon S Ganga HV Kluger J. Late gadolinium enhancement in patients with nonischemic dilated cardiomyopathy. Pacing Clin Electrophysiol. (2016) 39(7):731–47. 10.1111/pace.12873 27071516

[38] Kuruvilla S Adenaw N Katwal AB Lipinski MJ Kramer CM Salerno M. Late gadolinium enhancement on cardiac magnetic resonance predicts adverse cardiovascular outcomes in nonischemic cardiomyopathy. Circ Cardiovasc Imaging. (2014) 7(2):250–8. 10.1161/CIRCIMAGING.113.001144 24363358 PMC4007583

[39] Raman K S Nucifora G Leong DP Marx C Shah R Woodman RJ Long term prognostic importance of late gadolinium enhancement in first-presentation non-ischaemic dilated cardiomyopathy. Int J Cardiol. (2019) 280:124–9. 10.1016/j.ijcard.2019.01.018 30679073

[40] Krul SP Berger WR Smit NW van Amersfoorth SC Driessen AH van Boven WJ Atrial fibrosis and conduction slowing in the left atrial appendage of patients undergoing thoracoscopic surgical pulmonary vein isolation for atrial fibrillation. Circ Arrhythm Electrophysiol. (2015) 8(2):288–95. 10.1161/CIRCEP.114.001752 25673630

[41] Dendl K Koerber SA Kratochwil C Cardinale J Finck R Dabir M FAP And FAPI-PET/CT in malignant and non-malignant diseases: a perfect symbiosis? Cancers (Basel). (2021) 13(19):1–17. 10.3390/cancers13194946 PMC8508433 34638433

[42] Celiker-Guler E Ruddy TD Wells RG. Acquisition, processing, and interpretation of PET (18)F-FDG viability and inflammation studies. Curr Cardiol Rep. (2021) 23(9):124. 10.1007/s11886-021-01555-7 34269917

[43] Fathala A. Myocardial perfusion scintigraphy: techniques, interpretation, indications and reporting. Ann Saudi Med. (2011) 31(6):625–34. 10.4103/0256-4947.87101 22048510 PMC3221136

[44] Gupta S Ge Y Singh A Grani C Kwong RY. Multimodality imaging assessment of myocardial fibrosis. JACC Cardiovasc Imaging. (2021) 14(12):2457–69. 10.1016/j.jcmg.2021.01.027 34023250

[45] Cameli M Mondillo S Righini FM Lisi M Dokollari A Lindqvist P Left ventricular deformation and myocardial fibrosis in patients with advanced heart failure requiring transplantation. J Card Fail. (2016) 22(11):901–7. 10.1016/j.cardfail.2016.02.012 26952240

